# Characteristics of patients with ruptured abdominal aortic aneurysm who contacted out-of-hours primary care: a case-control study

**DOI:** 10.1186/s12245-025-00974-5

**Published:** 2025-09-02

**Authors:** Carline J. van den Dries, Dave A. Dongelmans, Maarten J. van der Laan, Sonja Oomkens, Eva Ouwendijk, Annelies Visser, Frans H. Rutten, Dorien L. M. Zwart

**Affiliations:** 1 Department of General Practice & Nursing Science, Julius Center for Health Sciences and Primary care, University Medical Center Utrecht, Utrecht University, P.O. Box 85500, Utrecht, 3508 AB The Netherlands; 2 Department of Intensive Care Medicine, Amsterdam UMC, Amsterdam Public Health, University of Amsterdam, Amsterdam, Netherlands; 3 Department of Surgery, University Medical Centre Groningen, Groningen, The Netherlands; 4 Founder of ‘Veiliger Zorg’, De Meern, The Netherlands; 5 Urgent Care Team, NEO Regional Primary Care Organization, Nijmegen, The Netherlands; 6 Department of Surgery, Amsterdam UMC, University of Amsterdam, Amsterdam, The Netherlands

**Keywords:** Ruptured abdominal aortic aneurysm, Triage, Primary care, Out-of-hours primary care services, Emergency medicine

## Abstract

**Background:**

Ruptured abdominal aortic aneurysm (rAAA) is rare but it is the second most frequently missed diagnosis reported as sentinel adverse event (‘calamity’) at out-of-hours services in primary care (OHS-PC).

We aimed to identify characteristics that could be useful for telephone triage of suspected rAAA at the OHS-PC.

**Methods:**

In a matched case-control study (1:4 ratio), we compared patients with a missed rAAA (cases) to patients with the same age and sex, and with similar entrance complaint (controls). Data were collected from OHS-PC triage call recordings that were re-assessed by researchers blinded to the case-control status. Patient and call characteristics were univariably assessed with conditional logistic regression analysis.

**Results:**

Twenty cases of missed rAAA between 2013 and 2023 were matched to 80 controls. 40% of the cases presented with abdominal pain, and 35% with back pain. Cases compared to controls more often had a pain onset < 12 h (odds ratio (OR) 15.2; 95%CI 1.9-123.8), reported more sweating (OR 10.1; 95% CI 1.2–86.9, *p* = 0.035), more often verbally expressed their concern (OR 13.6; 95%CI 3.0-61.3, *p* = 0.001), and more often called during the night (OR 3.8; 95% CI 1.1–12.7, *p* = 0.029).

**Conclusions:**

Recognition of rAAA at the OHS-PC remains challenging given its rare occurrence and lack of specific symptoms. Nevertheless, this case-control study identified factors that could be useful in triage of patients calling the OHS-PC with symptoms possibly indicating rAAA.

## Background

**Fig. 1:**
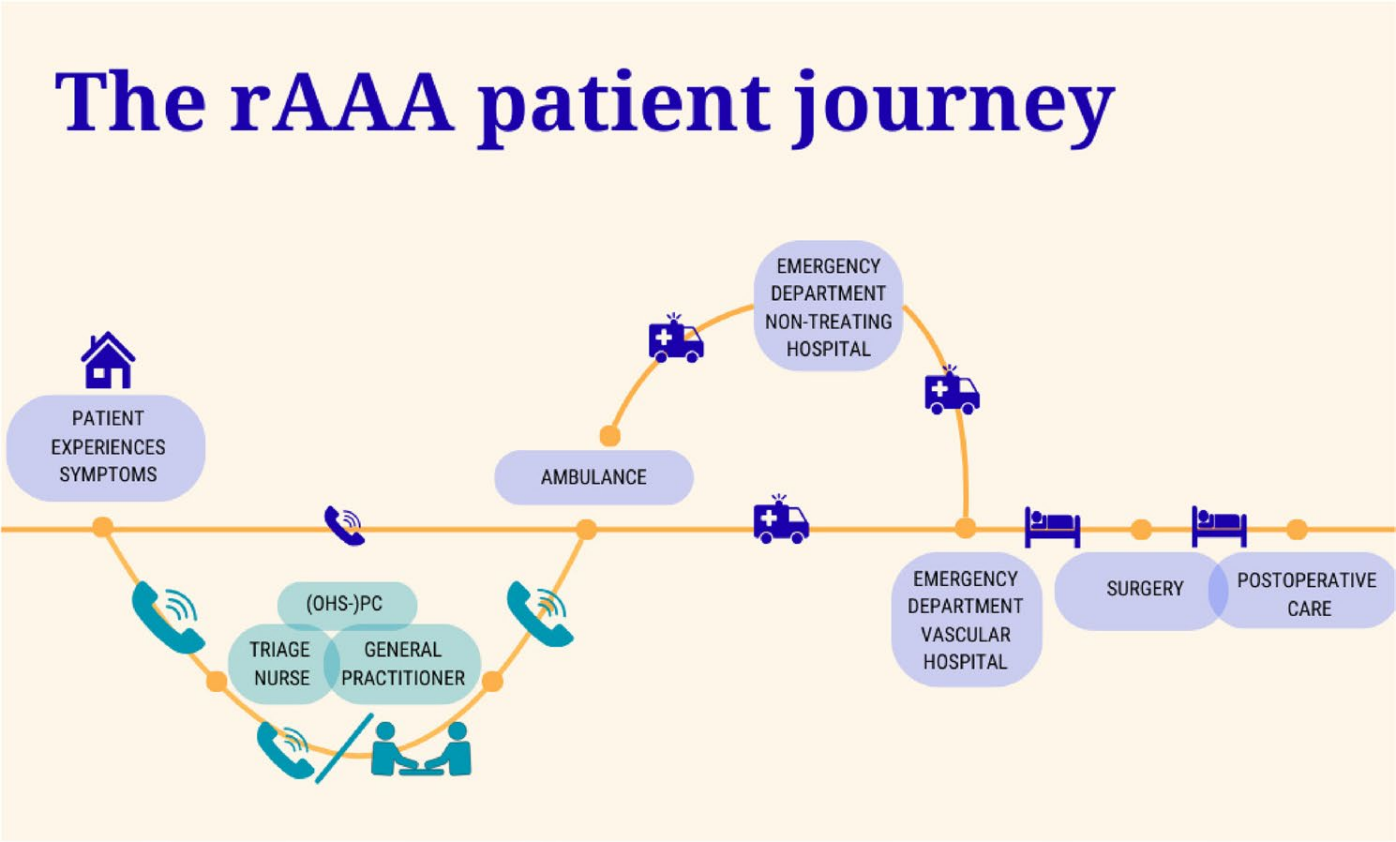
The disease trajectory of a patient with rAAA and caregivers and health settings involved OHS-PC: out of hours service in primary care. Turquoise part of journey: Telephone triage at the OHS-PC

A ruptured abdominal aortic aneurysm (rAAA) is a life-threatening condition requiring immediate surgical treatment. Overall mortality of rAAA patients is around 80–90%, when adding current in-hospital mortality around 30% (when patients reach the hospital alive) and an estimated 50% prehostipal mortality [1, 2]. To improve survival, early recognition is key. In The Netherlands and some other European countries, general practitioners (GPs) serve as gatekeepers of the healthcare system. During evening, night and weekend hours, telephone triage at out of hours services in primary care (OHS-PC) by triage nurses supervised by GPs is the first step in the care trajectory of patients in need of urgent GP care, although also direct access to the emergency department is possible by calling the emergency number 112. It is crucial that at the OHS-PC during this first conversation with the patient (or their representative), adequate information is collected to select the most appropriate urgency level (see Table 1), which further determines the next steps of the care trajectory (Fig. 1) and the time frame within which a patient needs to be seen by a medical professional.

**Table 1:** NTS levels of urgency

NTS urgency level	Definition	Respons time	Medical help
U0: Resuscitation	Loss of vital functions	Immediately	Ambulance
U1: Lifethreatening	Unstable vital functions	Within 15 min	Ambulance or immediate home visit by GP
U2: Emergent	Vital functions in danger	As soon as possible, at least within 1 h	Home visit by GP or consultation at OHS-PC
U3: Urgent	Possible risk of harm	Within 3 h	Home visit by GP or consultation at OHS-PC
U4: non-urgent	Marginal risk of harm	24 h	Consultation at OHS-GP or at own GP’s office
U5: non-urgent	No risk of harm	Advice at initial telephone call, not time related	Telephone consultation

Triage nurses in the Netherlands use a decision support tool called the “Netherlands Triage Standard” (NTS) to support them with urgency level allocation. The difficulty with rAAA is that signs and symptoms are often non-specific and mimic other conditions [3, 4]. Moreover, rAAA is a rare condition. In the Netherlands, with a population of 17,9 million people, 106 hospitals and 103 OHS-PCs across the country [5], in 2022 only 629 patients were hospitalized for rAAA (less than 2 patients per day in the Netherlands), and 240 people died from rAAA (< 1% of all cardiovascular deaths) [6]. In contrast, abdominal pain in adults was the third most common reason to contact the OHS-PC in 2023, with 16 calls per 1000 inhabitants (about 640 calls per day during out of hours) [7]. Scandinavian population based studies reported incidence rates of rAAA ranging from 4.3 to 21.3 per 100,000 person-years [8–10]. Such low incidence rates makes (telephone) triage a vulnerable and challenging process, with a high risk of delayed or missed rAAA diagnoses [11]. 

rAAA is after acute coronary syndrome the second most frequently missed diagnoses reported as a sentinel event or serious adverse event (SAE) at the OHS-PC in a Dutch study [12, 13], with an SAE defined as “an unintended or unexpected event related to the quality of care and resulting in death or a severe harmful event for the patient.” [14] SAEs are followed by root cause analysis, but this method is hampered by ‘hindsight bias’ [15]. A case-control design is ideal to learn from SAEs while avoiding hindsight bias.

We performed a case-control study to identify characteristics that are associated with missed or delayed rAAA.

## Methods

### Study design

We conducted a retrospective, matched case-control study, using data from triage calls from four different OHS-PCs. These OHS-PCs cover both rural, urban and suburban areas and are representative of OHS-PCs in the Netherlands. Calls were routinely recorded and archived for training, quality control and research purposes. As shown in Fig. 1, our study focused on triage of patients contacting the OHS-PC with symptoms suggestive of rAAA (turquoise route). We did not have data on rAAA patients for whom an ambulance was dispatched directly, before contact with a triage nurse.

### Study population

From four OHS-PCs together servicing a population of 1.7 million inhabitants, who were willing to share patient data and data on their ‘sentinel adverse events’, we retrieved data from August 2013 to July 2023 of all SAEs and their corresponding recordings of the triage calls concerning a missed diagnosis of rAAA. Missed rAAA was pragmatically defined as ‘when (i) the patient had died from a rAAA and when (ii) in the SAE analysis that was made and reported to the Dutch Inspectorate of Health it was evident that during triage this diagnosis had not been considered’. Controls were defined as patients calling the OHS-PC with similar age, sex and initially reported main symptom as cases, but whose contact did not end in an SAE. To increase statistical power, we matched four controls per rAAA case. When an exact age match was not possible, the ‘nearest neighbor’ closest in age was taken. Controls were sampled according to the ‘study base principle’, indicating that controls should not be sampled from the non-cases in order to avoid selection bias [16]. Therefore we sampled controls from a separate OHS-PC than the ones the cases came from. Controls were sampled from October 1st 2023 until March 10th 2024 from one OHS-PC.

### Data collection

Three researchers who were blinded to the case-control status of the patients collected the data: two of them collected data of the cases, and one researcher of the controls. Information on clinical characteristics (e.g., age, sex, symptoms, cardiovascular history) and call characteristics (e.g., time of calling, call duration, first person calling, consultation of a GP by the triage nurse, urgency level allocated) were collected. All information was retrieved from patient data that are routinely collected in clinical practice: (i) the triage call recordings, and (ii) the electronical medical records from the OHS-PC for the variables age, sex, entrance complaint, time of calling and call duration. We did not have data on the final diagnosis of the control patients, but we know they were not patients with a *missed* rAAA (i.e. cases) through verification with the OHS-PC. Hence, it is possible, but not very likely given the rare incidence, that patients with rAAA were included among controls. These patients should in a case-control study not be excluded because they belong to the domain of patients with possible rAAA [17, 18]. 

### Data analysis

We univariably assessed differences in characteristics between cases and controls, using conditional logistic regression analysis, the recommended analysis for matched case-control studies to control for matching factors [16, 19]. Conditional logistic regression analysis was done on those variables that we deemed clinically relevant for triage (based on our clinical knowledge and experience), in which a statistically significant difference could offer an opportunity for triage improvement. Odds ratios (ORs) with 95% confidence intervals (95%CI) were computed. A p-value < 0.05 was considered statistically significant. All analyses were performed in R, version 4.3.2.

### The NTS and missing data

The NTS is a decision support tool aimed at allocating the right urgency level during triage [20]. It is not a diagnostic tool. Based on the initial symptom (entrance complaint) that the patient reports, the NTS suggests additional questions that the triage nurse could ask. The triage nurse can also overrule the NTS suggestion. When certain answers indicate high urgency, the NTS generates a high urgency level straight away and, if the triage nurse agrees, the triage call can directly be finalized. This ‘closure’ may result in missing data on further questions. Additionally, data could be missing when a triage nurse did not ask a certain question, when it was asked but the caller did not give an answer, or when it is not applicable, e.g. ‘pain onset’ when the patient has no pain. We conducted a complete case analysis, as multiple imputation would likely not have increased validity given the limited number of cases.

## Results

A total of 20 rAAA cases were registered at three Dutch OHS-PCs during a decade (August 2013 - July 2023). These were compared to 80 matched controls. Baseline patient and call characteristics are shown in Table 2. Due to matching, cases and controls had similar age, sex and entrance complaint, most frequently being abdominal pain (40%) and back pain (35%).

**Table 2:** Baseline characteristics of patients calling the OHS-PC with symptoms possibly indicating rAAA

	Cases (*n* = 20)	Controls (*n* = 80)
Patient characteristics		
Median age (interquartile range) in years	66.0 (63.8–72.5)	66.5 (63.8–72.5)
Female sex (%)	5 (25)	20 (25)
Entrance complaint		
Abdominal pain	8 (40)	32 (40)
Back pain	7 (35)	28 (35)
Leg pain	1 (5)	4 (5)
Malaise	1 (5)	4 (5)
Dizziness	2 (10)	8 (10)
Arm pain	1 (5)	4 (5)
Pain onset < 12 h ago	17 (85)	31 (38.8)
* NA/not discussed*	*1 (5)*	*17 (21.2)*
Severe pain (VAS > 7 on a scale 0 to 10)	5 (25)	29 (36.2)
* NA/not discussed*	*14 (70)*	*34 (42.5)*
Shortness of breath	3 (15)	24 (30)
* Not discussed/unknown*	*10 (50)*	*19 (23.8)*
One or more ANS-related symptom	18 (90)	36 (45)
Ashen skin or pallor	3 (15)	13 (16.2)
* Not discussed/unknown*	*9 (45)*	*48 (60)*
Nausea or vomiting	11 (55)	21 (26.2)
* Not discussed/unknown*	*4 (20)*	*36 (45)*
Sweating	11 (55)	15 (18.8)
* Not discussed/unknown*	*8 (40)*	*39 (48.8)*
Dizziness/near fainting	3 (15)	15 (18.8)
* Not discussed/unknown*	*12 (60)*	*34 (42.5)*
History of abdominal aneurysm	1 (5)	3 (3.8)
* Not discussed/unknown*	*14 (70)*	*58 (72.5)*
History of cardiovascular disease	8 (40)	38 (47.5)
* Not discussed/unknown*	*10 (50)*	*34 (42.5)*
Use of cardiovascular medication	6 (30)	35 (43.8)
* Not discussed/unknown*	*7 (35)*	*27 (33.8)*
Caller verbally expresses concern	15 (75)	20 (25)
Call characteristics		
Time of calling		
Night (00.00–06.00 h)	7 (35)	11 (13.8)
Morning (06.00–12.00 h)	6 (30)	25 (31.2)
Afternoon (12.00–18.00 h)	2 (10)	18 (22.5)
Evening (18.00–00.00 h)	5 (25)	26 (32.5)
Median call duration in minutes (interquartile range)	8.8 (7.1–11.6)	10.9 (8.1–14.0)
Someone else called on behalf of the patient	8 (40)	42 (52.5)
Consultation of the supervising GP by triage nurse	11 (55)	47 (58.8)
Supervising GP takes over the call	1 (5)	14 (17.5)
Urgency level allocated		
U1	3 (15)	0 (0)
U2	5 (25)	30 (37.5)
U3	5 (25)	16 (20)
U4	3 (15)	6 (7.5)
U5	4 (20)	28 (35)
High urgency allocation (U1 or U2)	8 (40)	30 (37.5)

Data are presented as numbers (%) unless otherwise specified

The five different urgency levels are explained in Table 1

*NA* Not applicable, *VAS* Visual analogous scale, *GP* General practitioner, *ANS* Autonomous nervous system

Median age of cases was 66.0 (IQR 63.8–72.5) years, with a range from 58 to 80 years. In both cases and controls GPs were consulted by the triage nurse in about 55% of the calls, and a high urgency (U1 or U2) was allocated in 40% of cases and 37,5% of controls. However, the highest urgency level was more often given among cases (U1 in 15% of the cases versus 0% of the controls). Cases more often contacted the OHS-PC during the night than controls (35% vs. 13.8%).

Results of the univariable conditional logistic regression analyses are shown in Table 3. Among cases compared to controls, the onset of pain was more often less than 12 h ago (OR 15.2; 95%CI 1.9-123.8, *p* = 0.011). 90% of cases had one or more autonomous nervous system (ANS) related symptoms (pallor/ashen skin, nausea/vomiting, or sweating) versus 45% of controls; odds ratio 10.7 (95% CI 2.3–48.8, *p* = 0.002), mainly driven by sweating (55% vs. 18.8%, OR 10.1; 95% CI 1.2–86.9, *p* = 0.035).

Expressed concern (OR 13.6; 95% CI 3.0-61.3) and calling at night (OR 3.8; 95% CI 1.1–12.7) were also more common among cases than controls.

**Table 3:** Results of univariable conditional logistic regression analyses

	Cases (*n* = 20)		Controls (*n* = 80)		OR (95% CI)	*P*
Pain onset < 12 h ago	*n* = 19	17 (85)	*n* = 63	31 (39)	15.2 (1.9-123.8)	0.011
Shortness of breath	*n* = 10	3 (15)	*n* = 61	24 (30)	0.6 (0.1–2.8)	0.548
One or more ANS-related symptoms	*n* = 20	18 (90)	*n* = 80	36 (45)	10.7 (2.3–48.8)	0.002
Pallor or ashen skin	*n* = 11	3 (15)	*n* = 32	13 (16)	0.6 (0.1–2.6)	0.496
Nausea or vomiting	*n* = 16	11 (55)	*n* = 44	21 (26)	2.1 (0.7–6.6)	0.216
Sweating	*n* = 12	11 (55)	*n* = 41	15 (19)	10.1 (1.2–86.9)	0.035
Dizziness/near fainting	*n* = 8	3 (15)	*n* = 46	15 (19)	0.5 (0.0–5.0)	0.548
History of cardiovascular disease	*n* = 10	8 (40)	*n* = 46	38 (48)	1.0 (0.1–7.3)	0.974
Use of cardiovascular medication	*n* = 13	6 (30)	*n* = 53	35 (44)	0.2 (0.0-1.1)	0.064
Caller expresses concern	*n* = 20	15 (75)	*n* = 80	20 (25)	13.6 (3.0-61.3)	0.001
GP involved in triage		11 (55)		47 (59)	1.3 (0.4–4.2)	0.643
GP takes over the call		1 (5)		14 (18)	0.3 (0.04–2.8)	0.313
Calling during the night		7 (35)		11 (14)	3.8 (1.1–12.7)	0.029
Someone else called on behalf of the patient		8 (40)		42 (53)	0.6 (0.2–1.6)	0.325

Data are presented as numbers (%) unless otherwise specified. The columns with n = XX show the number of patients analyzed per variable

*GP* General practitioner, *ANS* Autonomous nervous system

## Discussion

In this matched case-control study, we compared recorded triage calls of 20 rAAA patients with 80 age- and sex-matched controls with similar symptomatology contacting the OHS-PC, to identify possible characteristics that could help improve triage of patients calling the OHS-PC with possible rAAA. Factors that were significantly more common at point of telephone triage among the 20 rAAA cases were (i) pain onset < 12 h ago (*p* = 0.011), (ii) sweating (*p* = 0.035), (iii) expressed concern by caller (*p* = 0.001), and (iv) calling at night (0.029).

### Comparison to existing literature

A similar matched case-control study has been previously done in the Netherlands among patients calling the OHS-PC with symptoms suggestive of acute coronary syndrome (ACS) [21]. Both ACS and rAAA are major cardiovascular events with degeneration as the underlying etiology. A major difference is that ACS is much more common than rAAA. In 2022, 33.769 patients were hospitalized with ACS in the Netherlands compared to 629 patients with rAAA [6, 22]. ACS has more specific symptoms than rAAA; nearly all patients have chest discomfort while rAAA patients may report more diverse symptoms such as abdominal pain (40%), back pain (35%), or in 25% either dizziness/near collapse, malaise, arm pain or leg pain. This implies much more mimics, that is, possible alternative underlying disorders in rAAA patients. This, together with the very low incidence of rAAA, makes correct suspicion of rAAA much more difficult compared to suspecting ACS in adults calling with chest discomfort. Moreover, in contrast to ACS, the very low numbers of rAAA in this setting does not allow for building a mulitivariable logistic regression model to develop a clinical prediction rule. Only the univariable relation between determinants and the outcome rAAA could be provided.

A few studies investigated which clinical factors were associated with misdiagnosis of rAAA [3, 4, 23]. These three hospital-based retrospective studies reported shock or collapse to be associated with a *decreased* risk of rAAA misdiagnosis. In the study by Smidfelt and colleagues, vomiting and shortness of breath were associated with an *increased* risk of rAAA misdiagnosis [23]. These associations were not present in our study, which can be clarified by differences in study populations.

### Clinical implications and suggestions for future research

rAAA is a rare condition that initially may present with non-specific symptoms, e.g., abdominal pain or back pain, which is followed by a fulminant course that in no time leaves only a small ‘window of opportunity’ for adequate triage, diagnosis, and treatment. We did not distinguish between symptomatic non-ruptured and ruptured rAAA because of similarity in symptoms, making distinction during triage impossible, and patients who eventually show to have symptomatic non-ruptured AAA should also be referred to the hospital with the highest urgency. Currently, the entrance complaints ‘abdominal pain adults’ and ‘back pain’ of the NTS generates questions to be asked by the triage nurse about the onset of pain, sweating, and whether there is a suspicion or medical history of AAA, but it does not consider expression of concern or time of calling. In line with other studies, expression of concern is an important determinant. We observed in a conversation analytic study that not exploring or addressing these concerns could lead to interactional difficulties in telephone triage of callers with chest discomfort [24]. In a Danish study on telephone triage at OHS-PC, it was shown that the caller’s subjective high “degree-of-worry” (DOW scale ranging 1–5) was associated with subsequent increased risk of hospital admission (adjusted OR 3.2 (2.0–5.0) and 3.1 (95%CI 2.0–5.0) for a DOW score 4 or 5, respectively) [25]. Patient concern is a ‘red flag’ for telephone triage conversation, and already easily triggers triage nurses in daily practice to increase the urgency level. However, because of its subjectivity concern is not always a red flag and therefore unfit to incorporate as a fixed item in the NTS-algorythm.

We want to stress the importance of asking during triage for autonomous nervous system related symptoms, notably sweating. Information on this item, according to our findings potentially suggestive for rAAA, was missing in 40% and 49% of cases and controls, respectively. Information on whether the patient had a history of AAA was also often missing (in 70% of all patients). As patients are not always aware of an AAA being diagnosed in the past (or able to answer this question correctly when calling the OHS-PC in severe pain), it is crucial that this information is made available for the triage nurse in the medical file of the OHS-PC.

Because the symptomatology of a rAAA is aspecific, a promising approach may be to improve early diagnosis with an imaging modality used by the ambulance or GP, e.g. a handheld ultrasound (point-of-care ultrasound, POCUS [26]). Especially because POCUS could also detect diagnostic mimics, e.g., urolithiasis or cholelithiasis. If POCUS could reliably show a normal aorta in the urgent primary care setting, targeted management of such a diagnosis could then be initiated by the GP, avoiding an emergency department visit. Efforts that improve collaboration in the ‘acute care chain’ could also significantly reduce the number of sentinel adverse events of missed rAAA diagnosis.

### Strengths and limitations

To the best of our knowledge, this is the first study that assessed determinants related to missed rAAA diagnosis in the OHS-PC setting using a case-control design. Moreover, data were collected during the first contact of the patient with the healthcare system, relistening the entire original triage call instead of scrutinizing notes or summarizing reports of healthcare workers that inherently only have a selection of the information a patient provides. Moreover, such notes are subjective interpretations of the healthcare professional. Another important strength of our study is that the researchers who collected the data were blinded to the case/control status of patients. Third, 20 SAE cases of missed rAAA is a rather large number considering its rare occasion.

There are also some limitations to consider when interpreting the results. Inherently to the routine care data and due to sometimes ‘early closing’ by the NTS system, we encountered missing data. It is common use when analysing routine care registry data to consider variables as absent if not recorded present in the electronic medical file, or -in our situation- in the re-listened telephone triage calls. We acknowledge this may result in some misclassification. However, such misclassification will not hamper the results if two groups are compared and the number of ‘uncertain’ missings is similarly distributed between groups for the relevant determinants as is shown in Table 2. Third, we did not have data on the final diagnosis of the control patients. Fourth, adequately matching control patients is challenging and it can be questioned how likely an rAAA diagnosis was in those control patients who had arm pain or leg pain as the entrance complaint, for example. However, there were in fact two rAAA cases who apparently presented with arm or leg pain as their initially reported (but not necessarily the only) symptom, further exemplifying that rAAA patients can present with non-specific symptoms, requiring not too closely matched controls. The approach we chose suits the way telephone triage works in daily practice: starting with the most urgent symptom the patient initially mentions and assigning the most appropriate urgency level without knowing the final diagnosis. We could only assess the triage at the OHS-PC and not the complete patient trajectory. It is of course possible that the cases ended up to be sentinel events not only because of suboptimal triage or urgency allocation, but also because of mistakes or a delay elsewhere in the patient trajectory.

Finally, there is possibly inter-observer variability, as data from cases (two researchers) and controls (one researcher) were collected by different researchers. Reassuring, however, is that 10% of the triage call recordings were scored in duplicate without revealing any inconsistencies.

## Conclusions

Triage, with recognition of and adequate urgency allocation in rAAA patients calling the OHS-PC, remains challenging given its rare occurrence and lack of disease-specific symptoms. Nevertheless, with this case-control study we identified factors that could be indicative for triage nurses: recent onset (heavy) abdominal or back pain with sweating in patients aged over 60, expressed concern, and/or calling at night should raise the suspicion for rAAA at the OHS-PC.

## Data Availability

The datasets analyzed during the current study are not publicly available due to privacy regulations, but are available from the corresponding author on reasonable request.

## Abbreviations

rAAA: Ruptured abdominal aortic aneurysm
OHS-PC: Out-of-hours services in primary care
GP: General practitioner
OR: Odds ratio
CI: Confidence interval
NTS: Netherlands Triage Standard
SAE: Serious adverse event
NA: Not applicable
VAS: Visual analogous scale
ANS: Autonomous nervous system
